# Feedback literacy among undergraduate dental students: A cross-sectional study across two institutions

**DOI:** 10.12669/pjms.42.2.12642

**Published:** 2026-02

**Authors:** Palwasha Babar, Ushna Malik

**Affiliations:** 1 Palwasha Babar, MDS, Associate Professor, Department of Paediatric Dentistry, Fatima Memorial Hospital College of Medicine and Dentistry, Lahore, Pakistan; 2 Ushna Malik, BDS, Demonstrator, Department of Medical Education, University College of Medicine and Dentistry, The University of Lahore, Lahore, Pakistan

**Keywords:** Attitudes, Dental education, Dental students, Feedback (Learning), Feedback literacy, Student feedback literacy scale (SFLS)

## Abstract

**Background & Objective:**

Feedback literacy (FL) is the ability to seek, interpret and apply feedback. It is critical for dental students’ clinical competence and professional growth. Despite its importance, empirical research on FL in dental education remains limited. The objective of the study was to assess FL among undergraduate dental students from two private dental institutions in Lahore using Student Feedback Literacy Scale (SFLS).

**Methodology:**

A cross-sectional study was conducted at two private dental colleges in Lahore from March to May 2025. A structured questionnaire including the validated SFLS was used to collect data. Participants included 245 Bachelor of Dental Surgery (BDS) students from first to final year while house officers were excluded. Statistical analyses included descriptive statistics, non-parametric tests for inferential analysis.

**Results:**

Internal consistency of the SFLS and its six subscales was strong, with Cronbach’s alpha values >0.80 across all domains. Participants demonstrated a high overall feedback literacy score (FLS), with a median of 3.79 (IQR=0.58). The eliciting domain score was lowest (median=3.75) indicating reluctance to seek feedback. Only 25.3% had received formal feedback training. Verbal feedback was the primary mode (51.8%). FLS differed significantly by perceived usefulness (p < 0.001) and by academic year (p = 0.011).

**Conclusions:**

Considerable variation was observed in feedback practices, including frequency, sources, and modes of feedback received. Dental students reported high FL but lower scores in the eliciting domain highlight the need for structured feedback training to support self-directed learning.

***Abbreviations:***

**FL:** Feedback Literacy, **FLS:** Feedback Literacy Score, **SFLS:** Student Feedback Literacy Scale.

## INTRODUCTION

Feedback is an integral part of health professions education as it plays a vital role in improving students’ learning and psychomotor skills.^1^ In dental education, it facilitates skill development, promotes student engagement, enhance student motivation and ensure safe and effective patient care.^2^ The traditional concept of the feedback process has evolved from being a unidirectional, teacher-centered approach to a bi-directional, student-centered approach, emphasizing students’ active, reciprocal involvement for a comprehensive learning experience.^3^ Carless and Boud^4^ argue that feedback can only enhance learning effectively when students possess the capabilities to not only understand and interpret feedback but also actively seek it out and translate it into actions, which they define as ‘feedback literacy’. Feedback literacy (FL) is underpinned by the premise that students act as active agents who take ownership of their learning, thereby engaging with feedback to enhance their study practices and achieve their personal educational objectives.

The framework of FL developed by Molly and Boud^5^ highlights the multilayered and interconnected competencies of FL, including feedback appreciation, evaluative judgment, affective regulation, the ability to implement change and recognizing standards to set meaningful learning goals. Therefore, when students do not acquire these competencies, they may fail to benefit even from high-quality feedback. This underscores the need to intentionally cultivate these capacities alongside effective feedback delivery processes.

A similar construct is feedback orientation, which refers to an individual’s attitudinal disposition or general willingness to seek, accept and use feedback.^6^ Feedback orientation focuses on learners’ attitudes and receptivity toward feedback, emphasizing enduring personal traits across situations. In contrast, FL encompasses the broader skills, understandings and dispositions needed to interpret and apply feedback, with distinct subcomponents that can be independently assessed, making it a more comprehensive and skill-based construct.

In dental education, students often engage with diverse and complex clinical feedback from multiple supervisors, requiring strong FL to effectively interpret and apply this information for meaningful learning and improvement.^7^ Despite advances in conceptual understanding, empirical research on FL remains scarce, with most existing studies in medical education relying primarily on qualitative methods.^8^,^9^ Its largely conceptual nature limits understanding of variations across populations and contexts. The Student Feedback Literacy Scale (SFLS) developed by Zhan,^10^ gives a quantitative assessment of FL. This assessment is essential to measure students’ feedback behaviors and attitudes, enabling educators to identify gaps and tailor strategies to improve FL among students.

Addressing this gap in the literature, the current study aims to assess FL among undergraduate dental students. The primary objective of the study was to assess FL among undergraduate dental students from two private dental institutions in Lahore. The secondary objectives included exploring factors that may influence FL, examining existing feedback practices and validating the SFLS within dental education context.

## METHODOLOGY

This cross-sectional study was conducted from March to May 2025 at two private dental institutes in Lahore, Fatima Memorial Hospital College of Medicine and Dentistry (Institution A) and University College of Medicine and Dentistry, The University of Lahore (Institution B).

### Ethical Approval:

The study adhered to the Declaration of Helsinki and follows STROBE guidelines for reporting. Ethical approval was obtained from the IRB of Fatima Memorial Hospital (Ref # FMH-06/03/2025-IRB-1629, Date: May 6, 2025), with informed consent taken and confidentiality maintained through anonymous responses. Undergraduate BDS students from first to final year who consented were included, while house officers, students on leave or those who declined consent were excluded. A sample size of 245 was calculated based on a 90% confidence level, 5% margin of error and a prior estimated preparedness rate of 19.8%.^1^ Participants were recruited via convenience sampling and data was collected through Google Forms shared on official student WhatsApp groups with follow-up reminders.

The study used a self-structured questionnaire with three sections: demographics (age, gender, academic year, background and recent scores), feedback practices (frequency, sources, modes, types and usefulness) and the 24-item SFLS by Zhan^10^ (used with permission from the author), covering six domains, eliciting, processing, enacting, appreciation, readiness and commitment, with four items in each domain. Each domain had four items, rated on a 5-point Likert scale (strongly disagree to strongly agree) to provide response variability and scale sensitivity. The original items were used without modification to maintain the integrity of the scale. The instructions to the participants included a clear definition of feedback while avoiding the term “feedback literacy” to reduce bias.

### Instrument Validation and Reliability:

Face validity was established by two experts in medical and dental education by using a structured validation checklist, based on Oducado’s Survey Instrument Validation Rating Scale.^11^ Rated on a binary scale with qualitative comments, their feedback affirmed the tool’s clarity, contextual relevance and alignment with research goals. Minor suggested revisions were incorporated. Questionnaire comprehension was assessed through pilot testing with three students, and additional guidance was added to items that required clarification. To assess construct validity, Exploratory Factor Analysis (EFA) was conducted to evaluate the underlying structure of the SFLS in our sample. As the original validation was based on Chinese university students, reassessing the scale within Pakistan’s distinct academic and cultural setting was important. Sampling adequacy for EFA was confirmed (KMO = 0.905), and Bartlett’s test of sphericity was significant (χ^2^ = 5152.19, df = 276, p < .001), indicating that the data were suitable for factor analysis. The analysis supported the original six-factor structure of the SFLS with acceptable item loadings. The six factors explained approximately 72% of the variance indicating strong construct validity. The internal consistency of the SFLS was assessed using Cronbach’s alpha on the same study sample. The overall scale demonstrated excellent reliability (α = 0.958). Domain-wise reliability was good to excellent: Eliciting α = 0.855, Processing α = 0.882, Enacting α = 0.910, Appreciation α = 0.905, Readiness α = 0.871, and Commitment α = 0.871.

### Statistical Analysis:

All analyses were performed using SPSS (v27) and RStudio (v4.4.3). As the study focused on assessing feedback literacy, data from both institutions were combined without inter-group comparisons. Descriptive statistics were used to summarize demographic variables. Feedback Literacy Scores (FLS) for the SFLS and its domains were reported as median and interquartile range (IQR). FLS across all six domains showed significant deviation from normality based on Shapiro-Wilk and Kolmogorov-Smirnov tests (all <.001). Given the non-normal distributions, non-parametric tests (Mann-Whitney U test for gender and Kruskal-Wallis test for other variables) were used for group comparisons. Where these tests were significant, Dunn’s post-hoc test with Bonferroni correction was applied. Correlations between FLS and background variables were examined using Spearman’s correlation. A p-value <0.05 was considered statistically significant for all inferential tests.

## RESULTS

A total of 245 undergraduate dental students, 137 (55.9%) from Institution-A and 108 (44.1%) from Institution-B participated in the study. The mean age was 21.2 years (SD±1.56), with a range from 18 to 24 years. Among the participants, 76.3% were female and 23.7% were males, which is representative of the gender profile in medical schools in Pakistan. Final year students had the highest representation and least by 1^st^ year. The details of the demographic variables of the participants are given in Table-I.

**Table-I:** The demographic details of the participants enrolled in the study.

Variable	Frequency (%)
***Gender***	
Male	58 (23.7)
Female	187 (76.3)
***Year***	
1^st^	35 (14.3)
2^nd^	47(19.2)
3^rd^	78(31.8)
4^th^	85(34.7)
***Educational Background***	
Matric-FSc	169(69)
O/A Levels	49(20)
Combination (O Levels ± FSc / Matric ± A Levels)	27(11)
***Percentage Score in Last Exam***	
50-60%	20(8.1)
61-70%	80(32.7)
71-80%	93(37.9)
81-90%	47(19.2)
91-100%	5(2.0)

Among the participants, 25.3% reported to have received formal training on feedback. The responses indicated that 23.7% ‘often’ receive the feedback, while 26.1% and 5.3% reported to ‘rarely’ and ‘never’ receive feedback respectively. Verbal feedback was reported to be the predominant mode of feedback (51.8%) followed by written and digital formats. The feedback was mostly received from clinical supervisors (37.4%) and teaching faculty (20.3%). 8.5% of the students engaged in self-assessment and seniors were cited as feedback providers by 18.5% of the participants. The majority of participants selected formative feedback as their primary experience. Regarding the perceived usefulness of the feedback, 41.2% found it to be ‘moderately useful’ and 32.6% and 6.9% found it to be ‘slightly’ and ‘not useful at all’. The details of the feedback-related practices and perceived usefulness reported by the participant are summarized in Table-II.

**Table-II:** Summary of Feedback-Related Practices, Sources, Modes and Usefulness Reported by Undergraduate Dental Students (*Multiple responses allowed).

Feedback Practices	Frequency (%)	Feedback Practices	Frequency (%)
***Frequency of receiving***		***Seeking from Faculty***	
Never Rarely	13(5.3) 64(26.1)	Never Rarely	13(5.3) 57(23.3)
Sometimes	100(40.8)	Sometimes	115(46.9)
Often	58(23.7)	Often	47(19.2)
Always	10(4.1)	Always	13(5.3)
Sources*		***Seeking from Peer/Others***	
Teaching Faculty	91(20.3)		16(6.5)
Clinical supervisors	168(37.4)	Never	59(24.1)
Classmates	69(15.4)	Rarely	96(39.2)
Seniors	83(18.5)	Sometimes	5(2.0)
Self-assessment (reflecting on your own performance)	38(8.5)	Often Always	21(8.5)
Mode*		***Perceived Usefulness***	
Written feedback	39(13.7)	Not useful at all	17(6.9)
Verbal feedback	147(51.8)	Slightly useful	80(32.6)
Digital/Online	98(34.5)	Moderately useful	101(41.2)
		Very useful	43(17.5)
		Extremely useful	4(1.6)
***Type***			
Formative	132(53.9)		
Summative	79(32.2)		
Formative ±Summative	34(13.9)		

Overall FLS were high across the cohort, with a median total score of 3.79 (IQR 0.58). The eliciting domain had the lowest median score, 3.75 with IQR 1.00, reflecting greater variability in students’ responses compared to other domains. Year-wise analysis demonstrated a modest upward trend in overall FLS, with the highest median scores observed in final-year students. Across domains, appreciation of feedback remained consistently high throughout all academic years. In contrast, eliciting feedback was lower in the first year, with gradual improvement in later years. The overall and year-wise domain scores (median and IQR) are given in Table-III.

**Table-III:** Overall and Year-Wise Median and IQR of Feedback Literacy Scores

Domain	Overall Score	1^st^ Year	2^nd^ Year	3^rd^ Year	4^th^ Year
Total Feedback Literacy Score	3.79 (0.58)	3.65 (0.25)	3.83 (0.52)	3.79 (0.62)	3.92 (0.58)
Eliciting	3.75 (1.00)	3.25 (1.00)	4.00 (1.00)	3.75 (0.75)	4.00 (1.00)
Processing	4.00 (0.75)	3.75 (0.50)	4.00 (0.62)	3.75 (0.75)	4.00 (0.75)
Enacting	4.00 (0.75)	3.75 (0.69)	4.00 (0.62)	4.00 (1.00)	4.00 (0.75)
Appreciation	4.00 (0.50)	4.00 (0.94)	4.00 (0.50)	4.00 (0.94)	4.00 (0.50)
Readiness	4.00 (1.00)	3.50 (0.75)	3.75 (0.62)	4.00 (0.94)	4.00 (1.00)
Commitment	4.00 (0.75)	3.75 (0.69)	3.75 (0.75)	4.00 (0.75)	4.00 (0.75)

The comparative analyses indicated statistically significant differences in FLS based on year of study (p = 0.011) and perceived usefulness of feedback (p = <0.001). Comparison with gender, educational background and feedback frequency were not statistically significance.

Post-hoc group analysis demonstrated that FLS differed significantly across perceived usefulness categories. Participants who perceived feedback as moderately useful had significantly higher FLS than those rating it as slightly useful (p = 0.04), and those perceiving feedback as very useful also demonstrated significantly higher FLS compared to the slightly useful group (p = 0.0002). All other pairwise comparisons were not statistically significant. Post-hoc analysis for year of study indicated that FLS differed significantly by year. Participants in the 4th year had significantly higher scores compared to 1st-year students (p = 0.0074). No other pairwise comparisons between years were statistically significant, indicating that differences were mainly between the first and fourth years.

Spearman correlation analysis revealed that FLS had weak positive associations with year of study, frequency of feedback, and perceived usefulness (ρ = 0.170, 0.176 and 0.245, respectively), while associations with gender and education were negligible.

## DISCUSSION

The current study highlights the trends of feedback practices at two private dental institutes. About one-third of the students reported to ‘never’ or ‘rarely’ receive feedback, which shows that consistent and timely feedback is lacking for a significant portion of learners, potentially limiting their opportunities for guided improvement and self-directed learning. The majority of students identified clinical supervisors and teaching faculty as their primary sources of feedback, while peers and senior students were less frequently cited. Incorporating peer feedback alongside traditional methods presents a valuable opportunity to overcome faculty and resource limitations.^12^ Recognizing peers and seniors as feedback sources can improve clinical training, a dimension underutilized in Pakistani dental education.

In our study, verbal feedback emerged as a major mode of feedback, which is consistent with the findings of the study by Leung et al.^13^ However, despite the increasing availability of technology, digital tools for feedback were notably underutilized. A recent study explored the use of a mobile application for feedback delivery and demonstrated encouraging results.^14^ This approach warrants further exploration within the context of dental education. Our study found that students mainly received formative feedback before final grading, with less feedback given after summative assessments. This pattern, where exam scores often serve as the sole feedback, mirrors international trends of limited post-assessment feedback.^13^ Such minimal and non-constructive summative feedback may increase student anxiety and negatively influence future performance,^15^ highlighting the need for targeted improvement.

Participants in the study demonstrated higher levels of FL, suggesting a greater tendency to value and actively engage with feedback to enhance their learning. This orientation reflects a positive shift from a focus on exam performance toward a commitment to meaningful growth and excellence in medical education.^16^ A recent study assessing students’ FL revealed findings similar to those in our research.^17^ The study reported higher scores in students’ attitudes toward feedback and the quality of feedback received, while scores were lower in areas such as the perceived importance of feedback and reactions to it. Although the study employed a different measurement tool, the Residency Education Feedback Level Evaluation in Clinical Training (REFLECT) scale, its results align closely with our observations. The minor differences between the two studies may be attributable to variations in educational level (residents vs. undergraduate students), clinical responsibilities, and institutional feedback cultures, which can influence how feedback is experienced and utilized. In contrast, SFLS used in our study offers a more comprehensive and multi-dimensional assessment by capturing multiple dimensions of FL in an integrated manner.

Our study found statistically significant differences in FL across the four academic years. This in accordance with the findings of Ansari and Usmani,^1^ who reported that senior clinical students perceived feedback as more valuable and geared toward improvement than their junior counterparts. Similarly, another study involving medical undergraduates identified developmental variations throughout the program, particularly in students’ understanding of feedback purposes, their ability to identify feedback and their perceptions of the credibility of those providing it.^18^ This may be attributed to differences in how feedback is emphasized within the curriculum or varying levels of exposure to clinical learning experiences. The FL pattern in our study reflects the dental program structure, rising in second year, dipping in third due to clinical transition and improving in final year with increased competence and faculty rapport.

In our study, the appreciation domain received high scores, reflecting students’ acknowledgment of the significance of feedback in their learning process. However, more than one-third of students rated the feedback they received as only ‘slightly useful,’ indicating a disconnect between their recognition of feedback’s value and their satisfaction with its quality. This low perceived usefulness of feedback among medical students resonates with findings from a previous study, in which 62% of students reported that feedback seldom offered constructive suggestions for improvement.^1^ The eliciting domain consistently received the lowest scores, reflecting students’ reluctance to seek feedback, possibly due to embarrassment or a weak feedback culture. Research shows feedback-seeking improves learning, emphasizing the need for clinical educators to foster supportive environments that encourage it.^19^ A qualitative study highlighted the key barriers to feedback-seeking in clinical settings, including an unsupportive culture, limited availability of supervisors and apprehension about receiving negative feedback.^17^ The authoritative culture prevalent in medical education, the demanding nature of clinical schedules and the presence of less approachable supervisors have also been identified as contributing factors to the reluctance to seek feedback.^14^ It has been found that students who receive early exposure to feedback-oriented learning are more receptive and proactive in using feedback.^20^ From an educational perspective, these findings highlight the need for structured FL interventions in dental education. The consistently lower scores in feedback eliciting suggest that feedback workshops should train students in how to seek feedback, ask clarifying questions and manage emotional responses to critique. Faculty-led workshops and mentoring systems can be designed to normalize feedback-seeking, encourage dialogue and provide students with opportunities to practice using feedback in both clinical and non-clinical settings. Additionally, mentoring by seniors or near-peers may help reduce hesitation and promote a supportive feedback culture within dental colleges.

### Strength & Limitation:

This study’s strengths include the use of a validated scale. Data was collected from two reputable dental institutions, enhancing relevance and generalizability. The validation of SFLS within context of health profession’s education allows its use to assess FL as a measurable construct, making it a valuable tool for future research across diverse medical and dental education settings. Limitations include potential response bias from self-reported data, the use of convenience sampling which may limit the generalizability of findings and a cross-sectional design that restricts the ability to assess changes in FL over time. Further qualitative researches are recommended to explore the underlying factors affecting FL.

## CONCLUSION

This study reveals that while dental students report generally high self-perceived FL, there is a notable gap in their willingness to actively seek feedback. The study offers valuable insights into an underexplored area in dental education to support the integration of structured FL training and mentoring systems within dental curricula to strengthen students’ capacity to seek and use feedback effectively. Such training can promote self-directed and lifelong learning, ultimately preparing students to become reflective and competent dental professionals.
